# Syndromic Inherited Retinal Diseases: Genetic, Clinical and Diagnostic Aspects

**DOI:** 10.3390/diagnostics10100779

**Published:** 2020-10-02

**Authors:** Yasmin Tatour, Tamar Ben-Yosef

**Affiliations:** Ruth & Bruce Rappaport Faculty of Medicine, Technion-Israel Institute of Technology, Haifa 31096, Israel; yasmin.t.90@gmail.com

**Keywords:** retina, inherited retinal diseases, syndrome

## Abstract

Inherited retinal diseases (IRDs), which are among the most common genetic diseases in humans, define a clinically and genetically heterogeneous group of disorders. Over 80 forms of syndromic IRDs have been described. Approximately 200 genes are associated with these syndromes. The majority of syndromic IRDs are recessively inherited and rare. Many, although not all, syndromic IRDs can be classified into one of two major disease groups: inborn errors of metabolism and ciliopathies. Besides the retina, the systems and organs most commonly involved in syndromic IRDs are the central nervous system, ophthalmic extra-retinal tissues, ear, skeleton, kidney and the cardiovascular system. Due to the high degree of phenotypic variability and phenotypic overlap found in syndromic IRDs, correct diagnosis based on phenotypic features alone may be challenging and sometimes misleading. Therefore, genetic testing has become the benchmark for the diagnosis and management of patients with these conditions, as it complements the clinical findings and facilitates an accurate clinical diagnosis and treatment.

## 1. Introduction

The retina is a multi-layered sensory tissue that lines the back of the eye. Its main function is the transduction of light energy into an electrical potential change, via a process known as phototransduction. The light-sensitive elements of the retina are the photoreceptor cells. The retina contains two types of photoreceptors, rods and cones. Rods (approximately 120 million in the human eye) are in charge of night vision, while cones (6 to 7 million in the human eye) are in charge of visual acuity and color vision. The highest cone concentration is found in the central region of the retina, known as the macula. Photoreceptors are highly compartmentalized cells, with the nucleus and other cellular organs located in the inner segment (IS), while the entire phototransduction machinery is included in the outer segment (OS).

Inherited retinal diseases (IRDs), which are among the most common genetic diseases in humans, define a clinically heterogeneous group of disorders, which cause visual loss due to improper development, dysfunction or premature death of the retinal photoreceptors [1]. IRDs are distinguished by several factors, including the type and location of affected cells and the timing of disease onset. The most common form of IRD is retinitis pigmentosa (RP) (also known as rod–cone dystrophy) [2]. Other IRD forms include cone/cone–rod dystrophy (CD/CRD) [3]; Leber congenital amaurosis (LCA) [4]; macular dystrophy (MD); and achromatopsia (rod monochromatism) [5], among others.

IRD is also one of the most genetically heterogeneous groups of disorders in humans, with over 260 genes identified to date (RetNet at https://sph.uth.edu/retnet/). It can be inherited as autosomal recessive (AR), autosomal dominant (AD) or X-linked (XL). Mitochondrial and digenic modes of inheritance have also been described. While in most cases of IRD the disease is limited to the eye (non-syndromic), over 80 forms of syndromic IRD have been described. Approximately 200 genes are associated with these syndromes (Table 1). In some cases of syndromic IRD, the retinal disease may be the presenting symptom and other systemic findings evolve during childhood, puberty or later on in life. In other cases, the first identifiable symptom of the syndrome is non-ocular and the retinal phenotype is revealed only later in life.

The topic of systemic diseases associated with IRDs has been reviewed before, including the description of some of these syndromes [6]. In the current review, we provide a comprehensive summary of the vast majority of syndromic IRD forms reported to date, for which the underlying gene/s have been identified (as listed in OMIM-Online Mendelian Inheritance in Man, https://www.ncbi.nlm.nih.gov/omim, and reported in the literature). We discuss different aspects, including the marked genetic heterogeneity of some of these syndromes, phenotypic overlap and diagnostic approaches.

## 2. Syndromic IRD Types

The majority of syndromic IRDs are recessively inherited and rare. Many, although not all, syndromic IRDs can be classified into one of two major disease groups: inborn errors of metabolism (IEM) and ciliopathies.

IEMs are genetic disorders leading to failure of carbohydrate metabolism, protein metabolism, fatty acid oxidation or glycogen storage. Many IEMs present with neurologic symptoms [9]. The retina develops from an embryonic forebrain pouch and is considered an extension of the brain. Therefore, neurodegeneration resulting from IEMs often involves retinal degeneration (RD) as well. Major forms of syndromic IRD that belong to the IEM group include congenital disorders of glycosylation (CDG) [10], neuronal ceroid lipofuscinoses (CLNs) [11], mucopolysaccharidoses (MPSs) [12], peroxisomal diseases [13] and more (Table 1).

Ciliopathies are a group of genetic diseases caused by mutations in genes associated with the structure and function of primary cilia. Primary cilia function as signaling hubs that sense environmental cues and are pivotal for organ development and function, and for tissue homeostasis. By their nature, cilia defects are usually pleiotropic, affecting more than one system [14]. Photoreceptor OSs are highly modified primary sensory cilia. The proximal end of the OS is linked to the cell body (i.e., the IS) via a connecting cilium which is structurally homologous to the transition zone of primary cilia [15]. Consequently, retinal pathogenesis is a common finding in ciliopathies. Other organs which are commonly affected in ciliopathies are the central nervous system (CNS), kidney, liver, skeleton and inner ear. Major forms of syndromic IRD that belong to the ciliopathy group include Bardet–Biedl Syndrome (BBS) [16], Joubert Syndrome (JBTS) [17], Usher Syndrome (USH) [18], Senior–Løken Syndrome (SLN) [19] and Alstrom Syndrome (ALMS) [20] (Table 1).

## 3. Genetic Heterogeneity in Syndromic IRDs

Over 80 forms of syndromic IRD have been described (Table 1). Most of these syndromes are caused by a single gene. However, 14 of 81 (17%) of the syndromes listed in Table 1 are genetically heterogeneous, and some of them are associated with multiple causative genes. The most genetically heterogeneous forms of syndromic IRD are three recessively inherited ciliopathies: BBS, JBTS and USH. The protein products of the genes associated with each one of these ciliopathies tend to form multi-protein complexes in the retina and in additional tissues, thus explaining the similar phenotypes caused by mutations in each of these genes.

BBS (prevalence of about 1/125,000) is characterized by a combination of RP, postaxial polydactyly (and other skeletal abnormalities), hypogonadism, renal disease, intellectual disability (ID) and truncal obesity [16]. Twenty-one causative genes have been reported to date (OMIM) (Table 1). Their protein products are involved in lipid homeostasis, intraflagellar transport, establishing planar cell polarity, regulation of intracellular trafficking and centrosomal functions. Eight of these genes encode for subunits of a protein complex, the BBSome, which is integral in ciliary as well as intracellular trafficking [21]. In the retina, the BBSome is required for photoreceptor OS formation and maintenance [22], as well as for retinal synaptogenesis [23].

JBTS (prevalence of 1/55,000–1/200,000) is characterized by a peculiar midbrain–hindbrain malformation, known as the molar tooth sign. The neurological presentation of JBTS includes hypotonia that evolves into ataxia, developmental delay, abnormal eye movements and neonatal breathing abnormalities. This picture is often associated with variable multiorgan involvement, mainly of the retina, kidney and liver [17]. RD has been reported in 38% of patients [24]. To date, 36 causative genes have been identified, all encoding for proteins expressed in the primary cilium or its apparatus (OMIM). Mutations in 20 of these genes (listed in Table 1) have been specifically associated with RD and additional ocular abnormalities (such as nystagmus and oculomotor apraxia). Ocular abnormalities have also been reported in patients with mutations in most other JBTS genes. However, since RD was not specifically reported in these patients, these genes are not listed in Table 1. Given the marked phenotypic heterogeneity found in JBTS patients, it is very likely that a retinal phenotype will be associated with these genes in the future, as additional patients are discovered.

USH (prevalence of 1–4/25,000) is characterized by the combination of RP and sensorineural hearing loss (HL). Based on the severity and progression of HL, age at onset of RP and the presence or absence of vestibular impairment, the majority of USH cases can be classified into one of three clinical subtypes (USH1-3). Eleven USH genes have been identified to date (OMIM) (Table 1). Their protein products are associated with a wide range of functions, including actin-binding molecular motors, cell adhesion, scaffolding and cellular trafficking. USH proteins form complexes and function cooperatively in neurosensory cells of both the retina and the inner ear (reviewed in [18,25]).

## 4. Phenotypic Overlap in Syndromic IRDs

When referring to syndromic IRD, phenotypic overlap is a common phenomenon, which can be divided into three groups, as detailed below:

### 4.1. Phenotypic Overlap between Different IRD Syndromes

**Figure 1:**
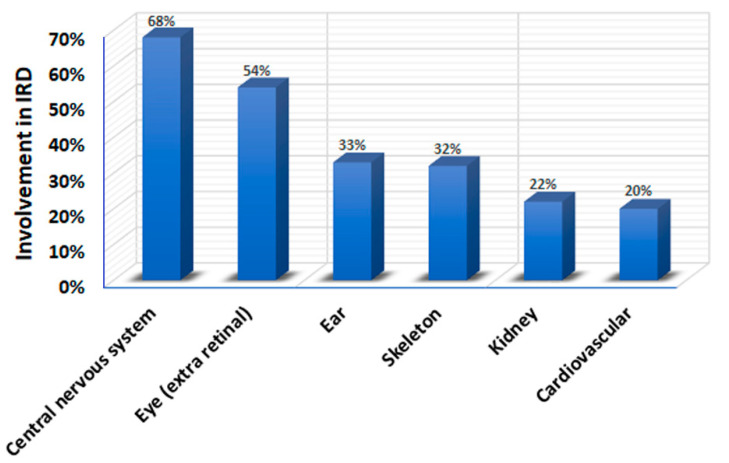
Systems and organs most commonly involved in syndromic IRDs.

Many types of syndromic IRD have a multi-systemic nature. Certain organs are commonly involved in syndromic IRDs. Specifically, CNS involvement (usually manifested as ID) is found in 68% of IRD syndromes (Table 1 and Figure 1), and over 80 genes are associated with the combination of IRD and ID [26] (Table 1). In addition to ID, the most common findings in syndromic IRD are extra-retinal eye abnormalities and ear, skeletal, renal and cardiovascular involvement (Figure 1). Most of these syndromes are phenotypically heterogeneous, with many patients exhibiting only some of the phenotypic features. These factors lead to a marked phenotypic overlap between different syndromes, and to a diagnostic challenge. For example, the combination of RD, ID, renal disease and skeletal abnormalities is found in numerous forms of syndromic IRD, including BBS, JBTS and ALMS, among others (Table 1). The combination of retinal abnormalities and HL as prominent symptoms is found in USH, as well as in CRD and HL 1 syndrome [27], Leber congenital amaurosis with early-onset deafness syndrome [28], Norrie disease [29], peroxisome biogenesis disorders, Refsum disease [30] and more (Table 1). These overlaps may often lead to diagnostic mistakes [27,31,32].

### 4.2. Syndromic Versus Non-Syndromic IRD Caused by the Same Genes

**Figure 2:**
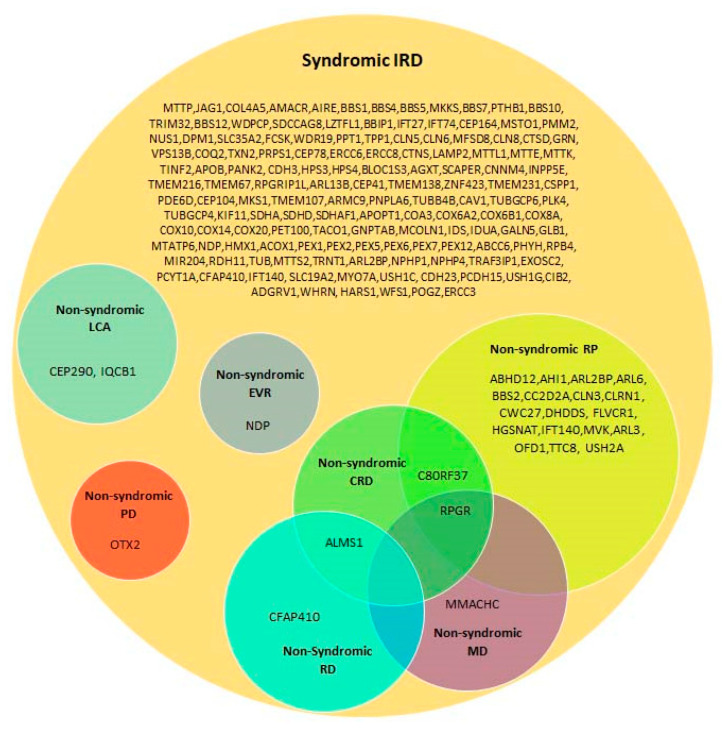
A Venn diagram showing the involvement of syndromic IRD genes in non-syndromic IRD phenotypes. CRD: cone–rod dystrophy; EVR: exudative vitreoretinopathy; LCA: Leber congenital amaurosis; MD: macular dystrophy; PD: pattern dystrophy; RD: retinal dystrophy; RP: retinitis pigmentosa.

Twenty-eight of the genes listed in Table 1 can cause both syndromic and non-syndromic IRD (Table 2 and Figure 2). In general, milder hypomorphic mutations in these genes are associated with non-syndromic IRD, while null mutations lead to the involvement of additional tissues. In addition, the involvement of additional genetic and environmental factors in the determination of the final phenotypic outcome cannot be excluded. A prominent example is the *USH2A* gene. Mutations in this gene are the most common cause of USH, and specifically of USH2 (RP with congenital, mild to moderate sensorineural HL) [33]. Moreover, *USH2A* variants are also one of the commonest causes of AR non-syndromic RP worldwide [34,35,36]. It appears that the specific combination of *USH2A* variants determines whether one has USH2 or non-syndromic RP [37,38,39]. In addition, RD is more severe in patients with *USH2A*-related USH2 than in patients with *USH2A*-related non-syndromic RP. However, the reason is not completely understood [38].

### 4.3. Co-Existence of Non-Syndromic IRD and Additional Non-Ocular Diseases

IRD is one of the most genetically heterogeneous groups of disorders in humans, and most cases of IRD are non-syndromic. Non-syndromic IRD may coincide with other genetic (and non-genetic) rare conditions, leading to a clinical suspicion or diagnosis of a syndrome. For example, co-occurrence of non-syndromic RP and non-syndromic HL in a family may appear as USH [71].

## 5. Diagnostic Challenges

Due to the high degree of phenotypic variability and phenotypic overlap found in syndromic IRD, as described above, correct diagnosis based on phenotypic features alone may be challenging and sometimes misleading. Therefore, genetic testing has become the benchmark for the diagnosis and management of patients with these conditions, as it complements the clinical findings and facilitates an accurate clinical diagnosis. Establishing a correct diagnosis is important for both the patients and their family members, for multiple reasons: it enables the understanding of the natural history course, and the prediction of disease prognosis; it aids in tailoring correct follow-up and treatment, including potential gene-targeted therapies [72]; it leads to a reduction in disease prevalence, by genetic screening and counseling in high-risk populations; it allows the patients to pursue prenatal counseling and reproductive planning; and it enables identification of novel disease genes and mechanisms.

The existence of common founder mutations in certain populations allows for quick and efficient mutation screening in affected individuals, based on the relevant phenotype and ethnic background. This is performed by PCR-based DNA amplification and Sanger sequencing, or by specifically designed assays. Some examples are common USH3A-, USH1F-, ML4- and BBS2-causative mutations found in the Ashkenazi Jewish population [73,74,75,76]; and USH3A- and MKS1-causative mutations found in the Finnish population [77,78]. Nevertheless, for most syndromic IRD patients worldwide, this strategy is not effective.

Currently, the most efficient approach for genetic diagnosis in monogenic diseases, including IRDs, is next-generation sequencing (NGS). NGS technologies facilitate the screening of the entire genome (whole genome sequencing, WGS); of all protein-coding regions (whole exome sequencing, WES); or of protein-coding regions of pre-determined panels of genes (targeted NGS, T-NGS) [79,80]. Since protein-coding regions comprise only 1–2% of the entire genome while harboring over 85% of variants causing Mendelian disorders, WES is still considered as the method of choice for genetic analysis, in both clinical and research settings. However, worldwide diagnostic yields of IRD patients by WES only range between 60% and 70% [36,81,82]. The missing mutations can be divided into four groups: (1) mutations located within exons, but missed due to technical issues, e.g., lack of coverage; (2) mutations located within covered exons, but missed due to limitations in data analysis and interpretation; (3) non-coding variants that may affect gene expression, mRNA stability, splicing and more; and (4) structural variants, such as large deletions, duplications and inversions, which are missed by WES. The latter two may be identified by WGS [34].

## 6. Summary and Conclusions

Over 80 forms of syndromic IRDs have been described, and approximately 200 causative genes identified. Due to the high degree of phenotypic variability and phenotypic overlap found in syndromic IRD, correct diagnosis based on phenotypic features alone is insufficient, and genetic testing has become the benchmark for the diagnosis and management of patients with these conditions. For most patients, molecular diagnosis should be based on NGS technologies. Currently, WES is the most popular approach for genetic analysis in patients with monogenic diseases, including IRDs. However, the continuous progress in both technical and bioinformatic aspects, as well as the reduction of costs, is already leading to a shift towards WGS as the method of choice.

## Figures and Tables

**Table 1:** Syndromic inherited retinal diseases (IRDs).

Syndrome (MIM/Reference)	Gene	Inheritance *	Main Ocular Phenotypes ^#^	Main Extra-Ocular Phenotypes ^¶^
Abetalipoproteinemia; ABL (#200100)	*MTTP*	AR	RP	Fat malabsorption, neurodegeneration, acanthocytosis
Aicardi Syndrome; AIC (#304050)	Xp22 abnormalities	XLD	Chorioretinopathy, OA, microphthalmia, optic nerve coloboma, cataract	Callosal agenesis, PGR, microcephaly, ID, skeletal anomalies, neoplasia
Alagille Syndrome 1; ALGS1 (#118450)	*JAG1*	AD	Iris stromal hypoplasia, posterior embryotoxon, microcornea, anomalous optic disc, peripapillary retinal depigmentation, chorioretinopathy	Liver disease, skeletal and renal involvement, characteristic facial features, ID, FTT
Alport Syndrome 1; ATS1 (#3010150)	*COL4A5*	XLD	Fleck retinopathy, cataract, myopia, corneal abnormalities	HL, renal disease
Alstrom Syndrome; ALMS (#203800)	*ALMS1*	AR	CRD, MD, cataract	DD, SS, obesity, HL, cardiac, skeletal, hepatic, renal and endocrine involvement
Alpha-Methylacyl-CoA Racemase Deficiency; AMACRD (#614307)	*AMACR*	AR	RP	Neurodegeneration
Autoimmune Polyendocrine Syndrome, Type I, with or without Reversible Metaphyseal Dysplasia; APS1 (#240300)	*AIRE*	AD, AR	RP, keratopathy, keratoconjunctivitis	Multiple autoantibodies, anemia, hepatic, gastrointestinal, dental, skin, hair and endocrine involvement, hypogonadism
Bardet–Biedl Syndrome; BBS (#209900, #615981, #600151, #615982, #615983, #605231, #615984, #615985, #615986, #615987, #615988, #615989, #615990, #615991, 615992, #615993, #615994, #615995, #615996, #617119, #617406) [7]	*BBS1*, *BBS2*, *ARL6*, *BBS4*, *BBS5*, *MKKS*, *BBS7*, *TTC8*, *PTHB1*, *BBS10*, *TRIM32*, *BBS12*, *MKS1*, *CEP290*, *WDPCP*, *SDCCAG8*, *LZTFL1*, *BBIP1*, *IFT27*, *IFT74*, *C8ORF37*, *CEP164*	AR	RP, strabismus, cataract	ID, SS, obesity, hypogonadism, renal disease, polydactyly
Cerebellar Atrophy with Pigmentary Retinopathy [8]	*MSTO1*	AR	RD	Cerebellar atrophy, ID, PGR
Congenital Disorder of Glycosylation; CDG (#212065, #617082, #613861, #608799, #300896)	*PMM2*, *NUS1*, *DHDDS*, *DPM1*, *SLC35A2*	AR	RP	FTT, microcephaly, ID, neurodegeneration, cardiac, hepatic, gastrointestinal, renal and hematological involvement
Congenital Disorder of Glycosylation with Defective Fucosylation 2; CDGF2 (#618324)	*FCSK*	AR	MD, OA, strabismus	FTT, ID, hypotonia, neurodegeneration, gastrointestinal anomalies
Cranioectodermal Dysplasia 4; CED4 (#614378)	*WDR19*	AR	RP	Skeletal anomalies, SS, respiratory, hepatic and renal involvement
Ceroid Lipofuscinosis, Neuronal; CLN (#256730, #204500, #204200, #256731, #601780, #610951, #600143, #610127, #614706)	*PPT1*, *TPP1*, *CLN3*, *CLN5*, *CLN6*, *MFSD8*, *CLN8*, *CTSD*, *GRN*	AR	RP, CRD, OA	Microcephaly, ID, neurodegeneration
Cohen Syndrome; COH1 (#216550)	*VPS13B*	AR	RD, OA, strabismus, high myopia	ID, DD, microcephaly, SS, obesity, skeletal, cardiac, hematological and endocrine involvement
Coenzyme Q10 Deficiency, Primary, 1; COQ10D1 (#607426)	*COQ2*	AR	RP	ID, cerebellar atrophy, HL, cardiac, hepatic, renal and muscular involvement
Combined Oxidative Phosphorylation Deficiency 29; COXPD29 (#616811)	*TXN2*	AR	RD, OA	Microcephaly, hypotonia, DD, ID, neurodegeneration
Charcot–Marie–Tooth Disease, X-linked recessive, 5; CMTX5 (#311070)	*PRPS1*	XLR	RP, OA	Peripheral neuropathy, HL
Cone–Rod Dystrophy and Hearing Loss 1; CRDHL1 (#617236)	*CEP78*	AR	CRD	HL
Cockayne Syndrome; CS (#216400, #133540)	*ERCC8*, *ERCC6*	AR	RD, OA, cataract, strabismus	IUGR, PGR, microcephaly, ID, neurodegeneration, HL, renal, skeletal and skin involvement
Cystinosis, Nephropathic; CTNS (#219800, #219900)	*CTNS*	AR	RD, corneal crystals	Renal disease, neurodegeneration, skeletal and endocrine anomalies
Danon Disease (#300257)	*LAMP2*	XLD	RD	Cardiac disease, myopathy, ID
Diabetes and Deafness, Maternally Inherited; MIDD (#520000)	*MTTL1*, *MTTE*, *MTTK*, mitochondrial DNA rearrangements	Mi	RD, MD, ophthalmoplegia	HL, cardiac and neurological anomalies, diabetes mellitus
Dyskeratosis Congenita, Autosomal Dominant 3; DKCA3 (#613990)	*TINF2*	AD	RD, blockage of lacrimal ducts	IUGR, SS, microcephaly, ID, HL, respiratory, skin, skeletal and hematological involvement, neoplasia
Hypobetalipoproteinemia, Familial, 1; FHBL1 (#615558)	*APOB*	AR	RP	Fat malabsorption, neurodegeneration, acanthocytosis
Hypobetalipoproteinemia, Acanthocytosis, Retinitis Pigmentosa and Pallidal Degeneration; HARP (#607236)	*PANK2*	AR	RP	Fat malabsorption, neurodegeneration, acanthocytosis
Hypotrichosis, Congenital, with Juvenile Macular Dystrophy; HJMD (#601553)	*CDH3*	AR	MD	Hypotrichosis
Hermansky–Pudlak Syndrome; HPS (#614072, #614073, #614077)	*HPS3*, *HPS4*, *BLOC1S3*	AR	Hypopigmentation of retina and choroid, foveal hypoplasia, nystagmus, iris transillumination	Skin and hair hypopigmentation, bleeding diathesis
Hyper-IgD Syndrome; HIDS (#260920)	*MVK*	AR	RP	Hematological anomalies, gastrointestinal and skeletal involvement, periodic fever
Hyperoxaluria, Primary, Type I; HP1 (#259900)	*AGXT*	AR	RD, OA	Renal disease, dental, cardiovascular and skin involvement, peripheral neuropathy
Intellectual Developmental Disorder and Retinitis Pigmentosa; IDDRP (#618195)	*SCAPER*	AR	RP, MD, cataract	ID, skeletal abnormalities, male sterility
Jalili Syndrome (#217080)	*CNNM4*	AR	CRD	Amelogenesis imperfecta
Joubert Syndrome; JBTS (#213300, #608091, #608629, #610188, #610688, #611560, #612291, #612285, #614464, #614465, #614844, #614970, #615636, #615665, #616781, #617121, #617562, #617622, #618161, #300804)	*INPP5E*, *TMEM216*, *AHI1*, *CEP290*, *TMEM67*, *RPGRIP1L*, *ARL13B*, *CC2D2A*, *CEP41*, *TMEM138*, *ZNF423*, *TMEM231*, *CSPP1*, *PDE6D*, *CEP104*, *MKS1*, *TMEM107*, *ARMC9*, *ARL3*	AR	RD, chorioretinal coloboma, optic nerve coloboma, microphthalmia, oculomotor apraxia, esotropia, ptosis	Brain structural anomalies, FTT, macrocephaly, ID, neurodegeneration, genitourinary, hepatic, respiratory and skeletal involvement
	*OFD1*	XLR		
Kearns–Sayre Syndrome; KSS (#530000)	Mitochondrial DNA deletions	Mi	RD, ophthalmoplegia	SS, microcephaly, neurodegeneration, cardiac, renal and endocrine involvement
Laurence–Moon Syndrome; LNMS (#245800)	*PNPLA6*	AR	Chorioretinal degeneration	ID, neurodegeneration, genitourinary abnormalities
Leber Congenital Amaurosis with Early-Onset Deafness; LCAEOD (#617879)	*TUBB4B*	AD	LCA	HL
Lipodystrophy, familial partial, type7; FPLD7 (#606721)	*CAV1*	AD	RD, cataract	Lack of facial fat, orthostatic hypotension, neurological and skin involvement
Methylmalonic Aciduria and Homocystinuria, cblC type; MAHCC (#277400)	*MMACHC*	AR	RP, CRD	FTT, microcephaly, ID, neurodegeneration, renal and hematological involvement
Mevalonic Aciduria; MEVA (#610377)	*MVK*	AR	RP, OA, cataract	FTT, DD, neurodegeneration, spleen, hepatic, skeletal, skin and hematological involvement
Microcephaly and Chorioretinopathy, autosomal recessive; MCCRP (#251270, #616171, #616335)	*TUBGCP6*, *PLK4*, *TUBGCP4*	AR	Chorioretinopathy, OA, microphthalmia, microcornea, cataract	IUGR, microcephaly, brain structural anomalies, DD, ID, neurodegeneration, SS
Microcephaly with or without Chorioretinopathy, Lymphedema or Mental Retardation; MCLMR (#152950)	*KIF11*	AD	Chorioretinopathy, myopia, hypermetropia, corneal opacity, microcornea, microphthalmia, cataract	Microcephaly, ID, neurodegeneration, lymphedema
Microphthalmia, Syndromic 5; MCOPS5 (#610125)	*OTX2*	AD	RD, microphthalmia, anophthalmia, optic nerve hypoplasia or agenesis, microcornea, cataract	Brain structural anomalies, hypotonia, pituitary dysfunction, DD, SS, cleft palate, abnormal genitalia, joint laxity
Mitochondrial Complex II Deficiency (#252011)	*SDHA*, *SDHD*, *SDHAF1*	AR	RD, OA, ptosis, ophthalmoplegia	SS, cardiac, skeletal, muscular and neurological involvement
Mitochondrial Complex IV Deficiency (#220110)	*APOPT1*, *COA3*, *COX6A2*, *COX6B1*, *COX8A*, *COX10*, *COX14*, *COX20*, *PET100*, *TACO1*	AR	RD, OA, ptosis	FTT, brain structural anomalies, ID, HL, cardiac, respiratory, hepatic, renal and muscular involvement
Mucolipidosis III alpha/beta; MLIII A/B (#252600)	*GNPTAB*	AR	RD, corneal clouding	Neurodegeneration, ID, SS, coarse facies, skeletal, cardiac and skin involvement
Mucolipidosis IV; ML4 (#252650)	*MCOLN1*	AR	RD, OA, corneal disease, strabismus	Microcephaly, ID, neurodegeneration
Mucopolysaccharidosis; MPS (#309900, #252930, #607014, #253000, #253010)	IDS	XLR	RP, ptosis, corneal clouding	Neurodegeneration, ID, SS, coarse facies, HL, skeletal, cardiac, respiratory, hepatic, gastrointestinal and skin involvement
	*HGSNAT*, *IDUA*, *GALN5*, *GLB1*	AR		
Nephronophthisis 15; NPHP15 (#614845)	*CEP164*	AR	LCA	Renal disease
Neurodegeneration with Brain Iron Accumulation 1; NBIA1 (#234200)	*PANK2*	AR	RD, OA, eyelid apraxia	Neurodegeneration, gastrointestinal, skeletal, skin and muscular involvement
Neuropathy, Ataxia and Retinitis Pigmentosa; NARP (#551500)	*MTATP6*	Mi	RP	Neurodegeneration, ataxia
Norrie Disease; ND (#310600)	*NDP*	XLR	Retinal dysgenesis, retinal dysplasia, OA, microphthalmia, vitreous atrophy, corneal opacities, iris atrophy, cataract	HL, ID, neurodegeneration
Oculoauricular Syndrome; OCACS (#612109)	*HMX1*	AR	RP, microphthalmia, microcornea, cataract, microphakia, sclerocornea, increased intraocular pressure	External ear abnormalities
Orofaciodigital Syndrome XVI; OFD16 (#617563)	*TMEM107*	AR	RD, oculomotor apraxia, ptosis	Facial anomalies, breathing abnormalities, polydactyly, hypotonia, ID, neurological anomalies
Oliver–McFarlane Syndrome; OMCS (#275400)	*PNPLA6*	AR	Chorioretinopathy, OA	SS, ID, neurodegeneration, obesity, male external genitalia abnormalities, endocrine anomalies
Peroxisomal Acyl-CoA Oxidase Deficiency (#264470)	*ACOX1*	AR	RD, OA, strabismus	Neurodegeneration, ID, HL, liver disease
Peroxisome Biogenesis Disorder; PBD (#214100, #614866, #601539, #234580, #614879, #266510)	*PEX1*, *PEX2*, *PEX5*, *PEX6*, *PEX7*, *PEX12*	AR	RD, OA, corneal clouding, cataract	FTT, neurodegeneration, ID, HL, dental, cardiac, hepatic, genitourinary and skeletal involvement
Posterior Column Ataxia with Retinitis Pigmentosa; AXPC1 (#609033)	*FLVCR1*	AR	RP, OA	Posterior column ataxia, neurodegeneration, gastrointestinal and skeletal involvement
Polyneuropathy, Hearing Loss, Ataxia, Retinitis Pigmentosa and Cataract; PHARC (#612674)	*ABHD12*	AR	RP, OA, cataract	Ataxia, neurodegeneration, HL
Pseudoxanthoma Elasticum; PXE (#264800)	*ABCC6*	AR	RD, MD, choroidal neovascularization	Skin lesions, cardiovascular disease, gastrointestinal and genitourinary involvement
Refsum Disease, classic (#266500)	*PHYH*	AR	RP	Neurodegeneration, ataxia, HL, anosmia, cardiac, skeletal and skin involvement
Retinal Dystrophy, Iris Coloboma and Comedogenic Acne Syndrome; RDCCAS (#615147)	*RPB4*	AR	RD, coloboma of the iris, displacement of the pupil, microcornea, cataract	Comedogenic acne
Retinal Dystrophy and Iris Coloboma with or without Cataract; RDICC (#616722)	*MIR204*	AD	RD, coloboma of the iris, congenital cataract	
Retinal Dystrophy, Juvenile Cataracts and Short Stature Syndrome; RDJCSS (#616108)	*RDH11*	AR	RD, juvenile cataracts	SS, DD, ID, dental anomalies
Retinal Dystrophy and Obesity; RDOB (#616188)	*TUB*	AR	RD	Obesity
Revesz Syndrome (#268130)	*TINF2*	AD	RD	IUGR, brain structural anomalies, neurodegeneration, ID, aplastic anemia, skin, hair and nail abnormalities
Retinitis Pigmentosa–Deafness Syndrome (#500004)	*MTTS2*	Mi	RP	HL
Retinitis Pigmentosa and Erythrocytic Microcytosis; RPEM (#616959)	*TRNT1*	AR	RP	Erythrocytic microcytosis and additional hematologic abnormalities
Retinitis Pigmentosa, Hypopituitarism, Nephronophtisis and mild Skeletal Dysplasia; RHYNS (#602152)	*TMEM67*	AR	RP	Hypopituitarism, renal disease, skeletal anomalies, HL
Retinitis Pigmentosa 82 with or without Situs Inversus; RP82 (#615434)	*ARL2BP*	AR	RP	Situs inversus, male infertility
Retinitis Pigmentosa with or without Skeletal Anomalies; RPSKA (#250410)	*CWC27*	AR	RP	SS, skeletal anomalies, ID
Retinitis Pigmentosa, X-linked and Sinorespiratory Infections, with or without Deafness (#300455)	*RPGR*	XL	RP	Recurrent respiratory infections, HL
Senior–Løken Syndrome; SLSN (#266900, #606996, #609254, #610189, #613615, #616307, #616629)	*NPHP1*, *NPHP4*, *IQCB1*, *CEP290*, *SDCCAG8*, *WDR19*, *TRAF3IP1*	AR	RP, LCA	Renal disease
Short Stature, Hearing Loss, Retinitis Pigmentosa and Distinctive Facies; SHRF (#617763)	*EXOSC2*	AR	RP, corneal dystrophy, glaucoma, strabismus	SS, facial anomalies, HL, neurodegeneration, DD, ID
Sideroblastic Anemia with B-cell Immunodeficiency, Periodic Fevers and Developmental Delay; SIFD (#616084)	*TRNT1*	AR	RP	Sideroblastic anemia, immunodeficiency, growth retardation, DD, periodic fever, HL, neurological, cardiac and renal involvement
Spondylometaphyseal Dysplasia with Cone–Rod Dystrophy; SMDCRD (#608940)	*PCYT1A*	AR	CRD	Skeletal anomalies, PGR
Spondylometaphyseal Dysplasia, Axial; SMDAX (#602271)	*CFAP410*	AR	RP, CRD, OA	Skeletal anomalies, respiratory disease, reduced sperm motility
Short-Rib Thoracic Dysplasia 9 with or without Polydactyly; SRTD9 (#266920)	*IFT140*	AR	RP	Skeletal anomalies, renal disease, ID
Thiamine-Responsive Megaloblastic Anemia Syndrome; TRMA (#249270)	*SLC19A2*	AR	OA, RD	Megaloblastic anemia, diabetes mellitus, HL
Usher Syndrome; USH (#276900, #276904, #601067, #602083, #606943, #614869, #276901, #605472, #611383, #276902, #614504)	*MYO7A*, *USH1C*, *CDH23*, *PCDH15*, *USH1G*, *CIB2*, *USH2A*, *ADGRV1*, *WHRN*, *CLRN1*, *HARS1*	AR	RP	HL, vestibular dysfunction
Wolfram Syndrome 1, WFS1 (#222300)	*WFS1*	AR	OA, RD	Diabetes mellitus, diabetes insipidus, HL, neurodegeneration, genitourinary and neurologic involvement
White–Sutton Syndrome, WHSUS (#616364)	*POGZ*	AD	RP, OA, cortical blindness	DD, characteristic facial features, hypotonia, HL, joint laxity, gastrointestinal anomalies
Xeroderma Pigmentosum, group B; XPB (#610651)	*ERCC3*	AR	RD, OA, micropathalmia	Neoplasia, skin anomalies, SS, microcephaly, HL, ID, brain structural anomalies, neurodegeneration

* AD, autosomal dominant; AR, autosomal recessive; Mi, mitochondrial; XL, X-linked; XLD, X-linked dominant; XLR, X-linked recessive. ^#^ CRD, cone–rod dystrophy; LCA, Leber congenital amaurosis; MD, macular dystrophy; OA, optic atrophy; RD, retinal dystrophy; RP, retinitis pigmentosa. ^¶^ DD, developmental delay; FTT, failure to thrive; HL, hearing loss; ID, intellectual disability; IUGR, intrauterine growth restriction; PGR, postnatal growth retardation; SS, short stature.

**Table 2:** Genes underlying both syndromic and non-syndromic IRDs.

Gene	Syndromic IRD	Non-Syndromic IRD (MIM)	Reference
*ABHD12*	PHARC	arRP	[40]
*AHI1*	JBTS3	arRP	[41]
*ALMS1*	ALMS	arCRD, arEORD	[42]
*ARL2BP*	RP with situs inversus	arRP (#615434)	[43,44]
*ARL3*	JBTS35	adRP (#618173)	[45]
*ARL6*	BBS3	arRP (#613575)	[46]
*BBS2*	BBS2	arRP (#616562)	[47]
*C8ORF37*	BBS21	arCRD, arRP (#614500)	[48]
*CC2D2A*	JBTS9, MKS6	arRP	[49]
*CEP290*	BBS14, JBTS5, MKS4, SLSN6	arLCA (#611755)	[50]
*CFAP410*	SMDAX	arRD with or without macular staphyloma (#617547)	[51,52]
*CLN3*	CLN3	arRP	[53]
*CLRN1*	USH3A	arRP (#614180)	[54]
*CWC27*	RPSKA	arRP (#250410)	[55]
*DHDDS*	CDG1BB	arRP (#613861)	[56,57]
*FLVCR1*	PCARP	arRP	[58]
*HGSNAT*	MPS3C	arRP (#616544)	[59]
*IFT140*	SRTD9 with/without polydactyly	arRP (#617781)	[60]
*IQCB1*	SLSN5	arLCA	[61]
*MFSD8*	CLN7	arMD (#616170), arRD	[62]
*MMACHC*	MAHCC	arMD	[63]
*MVK*	HIDS, MEVA	arRP	[64]
*NDP*	ND	XL EVR (#305390)	[65]
*OFD1*	JBTS10	XL RP (#300424)	[66]
*OTX2*	RD with pituitary dysfunction	adPD (#610125)	[67]
*RPGR*	RP, sinorespiratory infections and deafness	XL CRD (#304020), XL MD (#300834), XL RP (#300029)	[68]
*TTC8*	BBS8	arRP (#613464)	[69]
*USH2A*	USH2A	arRP (#613809)	[70]

ALMS: Alstrom syndrome; ar: autosomal recessive; ad: autosomal dominant; BBS: Bardet–Biedl syndrome; CDG: congenital disorder of glycosylation; CLN: ceroid lipofuscinosis neuronal; CRD: cone–rod dystrophy; EORD: early-onset retinal degeneration; EVR: exudative vitreoretinopathy; HIDS: hyper-IgD syndrome; JBTS: Joubert syndrome; LCA: Leber congenital amaurosis; MAHCC: methylmalonic aciduria and homocystinuria, cblC type; MD: macular dystrophy; MEVA: mevalonic aciduria; MKS: Meckel syndrome; MPS: mucopolysaccharidosis; ND: Norrie disease; PCARP: posterior column ataxia with retinitis pigmentosa; PD: pattern dystrophy; PHARC: polyneuropathy, hearing loss, ataxia, retinitis pigmentosa and cataract; RD: retinal dystrophy; RP: retinitis pigmentosa; RPSKA: retinitis pigmentosa with skeletal anomalies; SLSN: Senior–Løken syndrome; SMDAX: spondylometaphyseal dysplasia axial; SRTD: short-rib thoracic dysplasia; USH: Usher syndrome; XL: X-linked.
